# Variability in the Capacity to Produce Damage-Induced Aldehyde Green Leaf Volatiles among Different Plant Species Provides Novel Insights into Biosynthetic Diversity

**DOI:** 10.3390/plants9020213

**Published:** 2020-02-06

**Authors:** Jurgen Engelberth, Marie Engelberth

**Affiliations:** Department of Biology, University of Texas at San Antonio, One UTSA Circle, San Antonio, TX 78249, USA; Jengelberth@gmx.net

**Keywords:** green leaf volatiles, (*E*)-2-hexenal, (*Z*)-3-hexenal, hexanal, plant damage, plant volatiles, plant defense, plant species, release pattern

## Abstract

Green leaf volatiles (GLVs) are commonly released by plants upon damage, thereby providing volatile signals for other plants to prepare against the major causes of damage, herbivory, pathogen infection, and cold stress. However, while the biosynthesis of these compounds is generally well understood, little is known about the qualities and quantities that are released by different plant species, nor is it known if release patterns can be associated with different clades of plants. Here, we provide a first study describing the damage-induced release of major GLVs by more than 50 plant species. We found major differences in the quantity and quality of those compounds between different plant species ranging from undetectable levels to almost 100 µg per gram fresh weight. We also found major shifts in the composition that correlate directly to the quantity of emitted GLV. However, we did not find any major patterns that would associate specific GLV release with distinct clades of plants.

## 1. Introduction

In recent years, green leaf volatiles (GLV) were extensively characterized for their important role in the plant defense response against biotic and abiotic stresses, including insect herbivory and pathogen infection, as well as cold, heat, and salt stress [1,2,3,4,5,6,7,8,9]. Damaged plants almost instantly release GLV in large quantities, making them ideal chemical messengers for neighboring plants to prompt their defense responses [1,2,3]. However, while the direct protective activity of these compounds was always considered to be low, the priming of these responses appeared to be their major function resulting in an enhanced and, thus, more effective defense response when under actual attack. This GLV-induced priming was first described for maize (*Zea mays*) [10], but was since confirmed for many other plant species not only for defenses against herbivores and pathogens [11,12,13,14], but also against abiotic stresses [4,5,6,7,8].

**Figure 1:**
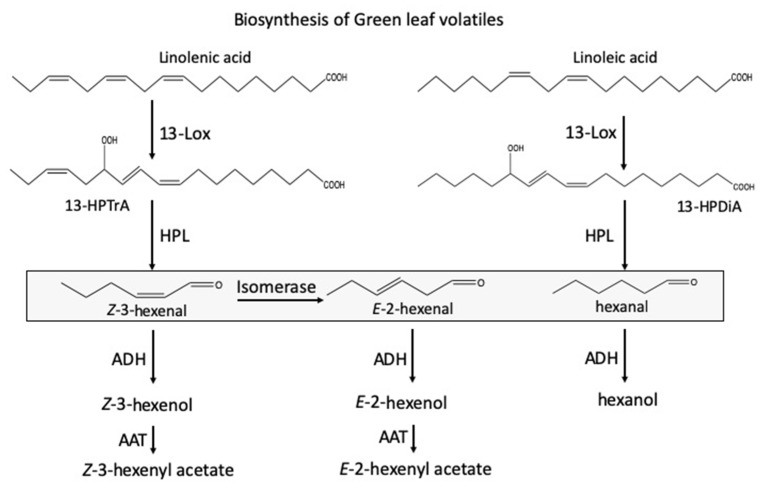
Biosynthetic pathways leading to green leaf volatile production in plants. A 13-lipoxygenase (13-LOX) catalyzes the addition of molecular oxygen at position 13 in linolenic acid and linoleic acid to form 13-hydroperoxy octadecatrienoic and 13-hydroperoxy octadecadienoic acid (13-HPTrA and 13-HPDiA). These oxygenated fatty acids are then converted to (*Z*)-3-hexenal and hexanal, as well as 9-(*Z*)-traumatin, by hydroperoxide lyase (HPL). An isomerase converts (*Z*)-3-hexenal into (*E*)-2-hexenal. (*Z*)-3-Hexenal and (*E*)-2-hexenal can be reduced to (*Z*)-3-hexenol and (*E*)-2-hexenol, respectively, by alcohol dehydrogenase(s) (ADH). (*Z*)-3-Hexenol and (*E*)-2-hexenyl acetate can further be converted to (*Z*)-3-hexenyl acetate and (*E*)-2-hexenyl acetate by alcohol acyltransferase(s) (AAT). Boxed compounds are those produced mainly by damaged plant tissue.

The biosynthetic pathway leading to the production of GLV is generally well understood [15,16,17,18] (Figure 1). GLVs are fatty acid-derived products formed from linolenic acid and linoleic acid, which serve as substrates for a pathway-specific 13-lipoxygenase (for maize LOX10 [19]). These fatty acids can be used as a substrate in the form of free fatty acids, but also when still conjugated to a lipid. The resulting 13-hydroperoxy C18 fatty acid is then cleaved by the enzyme hydroperoxide lyase (HPL), producing (*Z*)-3-hexenal (Z-3-HAL) (from 18:3 fatty acids) or hexanal (from 18:2 fatty acids), as well as 12-oxo-(*Z*)-9-decenoic acid, the natural precursor of traumatin. Recently, isomerases were identified and characterized from bell pepper (*Capsicum annuum*) and cucumber (*Cucumis sativus*) that convert Z-3-HAL into (*E*)-2-hexenal (E-2-HAL) [20,21]. However, while the gene was identified in several other plant species, many do not seem to have a functioning isomerase [20]. Both isomers can further be processed by hexenal reductase [22] and acetylation by an alcohol acyltransferase (AAT) [23] into the remaining C6-components like (*Z*)-3- and (*E*)-2-hexenol (Z-3-HOL, E-2-HOL) and (*Z*)-3- and (*E*)-2-hexenyl acetate (Z-3-HAC, E-2-HAC), respectively. While damaged cells produce mainly the aldehydes, neighboring intact cells are necessary to convert these into the corresponding alcohols and esters [24]. GLVs are almost immediately released locally from damaged tissue [15,16,17,18], but can also be produced systemically in response to herbivore damage [25]. Some plants emit GLV in a diel cycle with maximum emissions reported at the beginning of the scotophase, where this effect was found to be dependent on the availability of oxygen [26,27].

To date, little is known about how exactly plants regulate the release of GLV after damage. The key enzymes for the biosynthesis of GLV are LOX and HPL [15,16,17,18]. However, they seem to differ significantly in their location and how they are regulated. For example, some HPLs were found in chloroplasts associated with the thylakoid membranes [28], while others were located in the envelope membrane system [29,30,31]. Likewise, LOX enzymes can be localized in different compartments at the cell and organelle level as well [32,33,34]. The pH dependency of these enzymes can also vary significantly among different plant species. Most HPLs, for example, were found to be active at acidic pH levels [29,35,36,37]. This corresponded well with the pH of damaged plant tissue, which is around 5–6. In contrast, HPLs from spinach (*Spinacia oleracea*) and other plants were found to be most active at basic pH [38,39]. Furthermore, the 13-LOX (Lox10) in maize responsible for the production of GLV has a pH optimum in the basic range [40]. Yet, there is generally very little information available about the pH dependency of HPL and LOX in other plants. Moreover, while isomerases were identified [20,21], it is yet unclear what role they play in the damage-induce production of GLV.

Aside from variations in localization and pH dependency, substrate availability appears to be a further important factor that may regulate the production of GLV upon damage. However, little is in fact known about the distribution and quantities of hydroperoxy fatty acids in the plant chloroplast, especially when it comes to different plant species. This may have significant consequences for the production of GLV. For example, should hydroperoxyl fatty acids be readily available upon damage, HPL could instantly utilize these to produce Z-3-HAL. If 13-LOX enzymes have to produce these hydroperoxy fatty acids first, however, a delayed and/or reduced production of GLV could be expected.

Based on the available information about the regulation of GLV biosynthesis after damage, it becomes clear that no uniform picture exists that can be applied to different plant species. Additionally, although the general pathway leading to the production of GLV seems to be well characterized, how plants utilize their respective set-up of enzymes is still unknown. To gain further insights into the process of damage-induced GLV release, we initiated the study described here to firstly obtain information on the capacity of different plant species to produce GLV. This is of critical importance when performing meaningful experiments with the respective plant, which is always a matter of controversy. Furthermore, in light of recent discoveries on how insect herbivores suppress the production of GLV [41,42,43,44], knowledge about the amounts of GLV that can be produced by the respective plant species is essential in assessing the effectiveness of this suppression. We analyzed the composition of those GLVs with focus on the aldehydes (Z-3-HAL, E-2-HAL, and hexanal), mainly because other GLVs downstream are not produced by damaged tissue since they require intact cells for their biosynthesis [24]. This information may become important when analyzing the effectiveness of individual GLVs in a natural and laboratory context. The results show a complex pattern of damage-induced GLV production that varies tremendously among different plant species, leading to the conclusion that individual approaches to different plant species should be used when analyzing specific responses to biotic and abiotic stresses.

## 2. Results

### 2.1. Quantities and Qualities of Damaged-Induced GLV Vary Significantly among Different Plant Species

**Figure 2:**
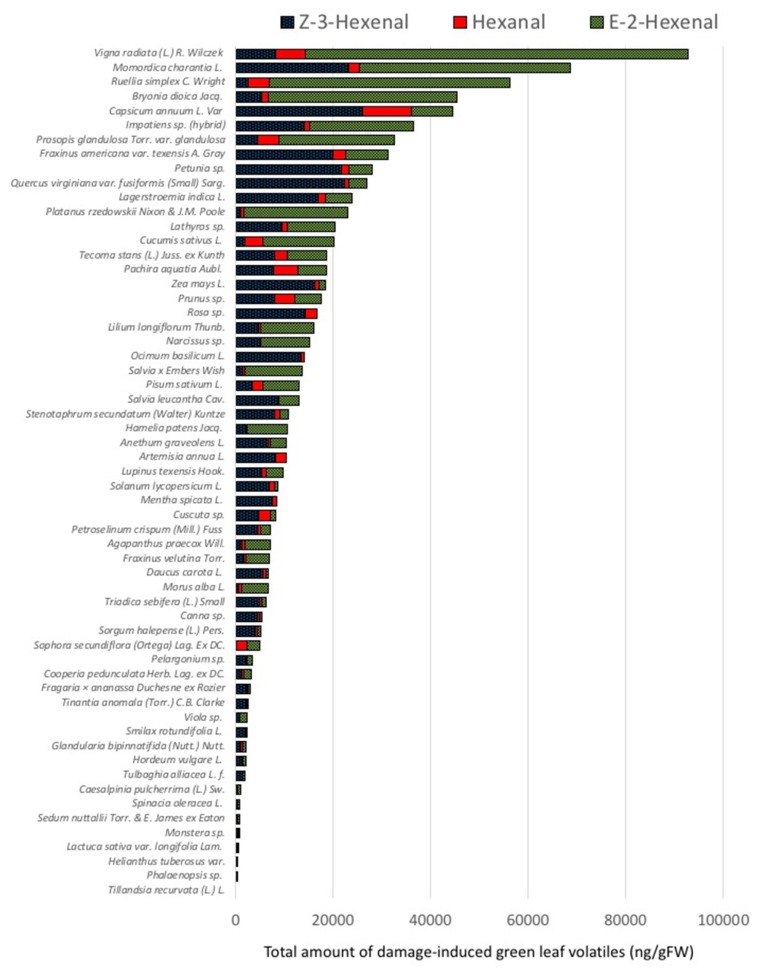
Damage-induced biosynthesis of green leaf volatiles (GLVs) from different plant species. Shown are amounts for (*Z*)-3-hexenal, hexanal, and (*E*)-2-hexenal in ng/g fresh weight (FW). Results for each plant are averages from at least three biological replicates. Data including standard deviation are shown in Table S1 (Supplementary Materials).

We focused our analysis on the three major compounds (Z-3-HAL, E-2-HAL, and hexanal) that are released by plant upon damage. Since other GLVs like hexenol and hexenyl acetate require intact cells for their biosynthesis, we did not monitor those compounds. A detailed list with average amounts and standard deviations of Z-3-HAL, E-2-HAL, and hexanal from all plant species analyzed for this study is also shown in Table S1 (Supplementary Materials). The total amounts of GLV emitted from damaged leaf tissues varied significantly, ranging from undetectable levels in *Tillandsia recurvata* to 92,850 ± 18,478 ng/g fresh weight (FW) in *Vigna radiata* leaves (Table S1, Supplementary Materials; Figure 2). *Zea mays* as our model plant produced 18,337 ± 6167 ng/g FW and was, as such, the highest producer of GLV among the monocot plants. Interestingly, spinach (*Spinacea oleracea*) and lettuce (*Lactuca sativa*), two major food plants, were among the lowest emitters of GLV with 913 ± 331 ng/g FW and 644 ± 195 ng/g FW, respectively, while green bell pepper (*Capsicum annuum*) with 44,464 ± 9998 ng/g FW was among the highest producers of GLV. Among the trees used for this study, we found the local mesquite tree (*Prosopis glandulosa*) to be the highest producer of GLV (32,600 ± 3378 ng/g FW). On the other side of the spectrum, mountain laurel (*Sophora secundiflora*), a local small tree with evergreen leaves, was the lowest producer of GLV (4844 ± 507 ng/g FW).

The first volatile compound produced in the biosynthetic pathway of GLV is Z-3-HAL for linolenic acid-derived GLV and hexanal for linoleic acid-derived GLV. As can be seen in Figure 2, hexanal is mostly a minor compound, ranging in its emission from 99 ± 14 ng/g FW in *Helianthus tuberosus* to 10,022 ± 3659 ng/g FW in *Capsicum annuum*, while *Tillandsia recurvata* and *Phalaenopsis* sp. showed zero emissions for this compound. Z-3-HAL, on the other hand, ranged from 281 ± 101 ng/g FW in *Phalaenopsis* sp. up to 26,034 ± 4777 ng/g FW in *Capsicum annuum*. While some plants did not produce detectable levels of E-2-HAL in our assays, damage-induced production in other plants ranged from 149 ± 38 ng/g FW (*Monstera* sp.) up to 78,485 ± 24,399 ng/g FW in *Vigna radiata* leaves, and it appears as if E-2-HAL is the major compound in the highly emitting plants. Aside from *Vigna radiata*, particularly high levels of E-2-HAL were found in the members of the cucurbitacea family including *Momordica charantia* (43,236 ± 14,777 ng/g FW), *Bryonia dioica* (38,696 ± 7502 ng/g FW), and *Cucumis sativa* (14,524 ± 1137 ng/g FW), as well as in *Ruellia simplex* (49,369 ± 5967 ng/g FW).

**Figure 3:**
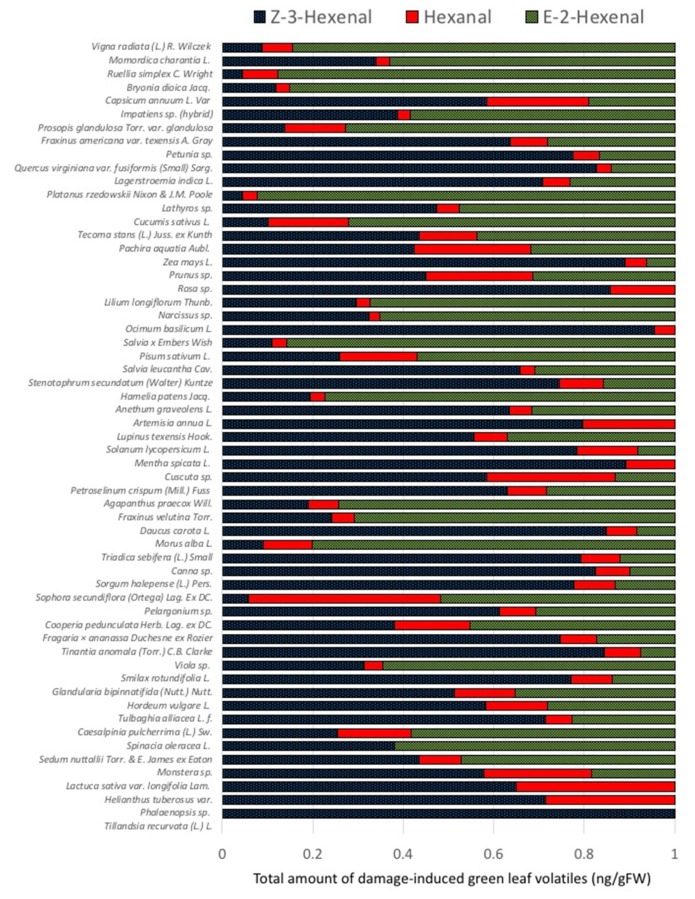
Proportions of GLVs emitted from damaged leaf tissue of different plant species. Shown is the relative abundance for (*Z*)-3-hexenal, hexanal, and (*E*)-2-hexenal as a percentage. Plants are organized as shown in Figure 1. Results for each plant species are averages from at least three biological replicates.

Results presented in Figure 2 suggest that E-2-HAL is the major GLV produced, particularly by those highly emitting plants including *Vigna radiata*, *Momordica charantia*, *Bryonia dioica*, *Cucumis sativa*, and *Ruellia simplex*. To further analyze the proportions of individual GLV among different plant species, we normalized the data by calculating the relative abundance of these compounds for each plant (Figure 3).

Plants were listed similar to Figure 2 with the highest overall emitter on top. While the highest emitting plants including *Momordica charantia*, *Vigna radiata*, *Bryonia dioica*, and *Ruellia simplex* seem to have higher proportions of E-2-HAL ranging from 63% to 87%, other high emitters like *Capsicum annuum*, *Lagerstroemia indica*, *Petunia* sp., and *Quercus virginianan* had higher proportions of Z-3-HAL ranging from 58% to 82%. *Capsicum annuum* and other members of the Solanaceae family showed relatively low proportions of E-2-HAL. This is surprising since the isomerase responsible for the conversion of Z-3-HAL to E-2-HAL was first identified in this family [20]. However, recently, an isomerase was also characterized from cucumber, which is in accordance with the data presented herein for members of that family. *Zea mays*, on the other hand, a plant we studied for years with regard to GLV emissions and responses [4,10], belongs to those plants with a high proportion of Z-3-HAL (89%). However, overall, it must be stated that individual proportions of GLV seem to vary significantly among the different plant species analyzed for this study.

### 2.2. Correlation Analysis

To gain further insights into the correlation between individual GLV and amounts, we performed correlation analyses. Firstly, we analyzed the correlation between amounts of individual compounds with the total amount of GLVs released.

**Figure 4:**
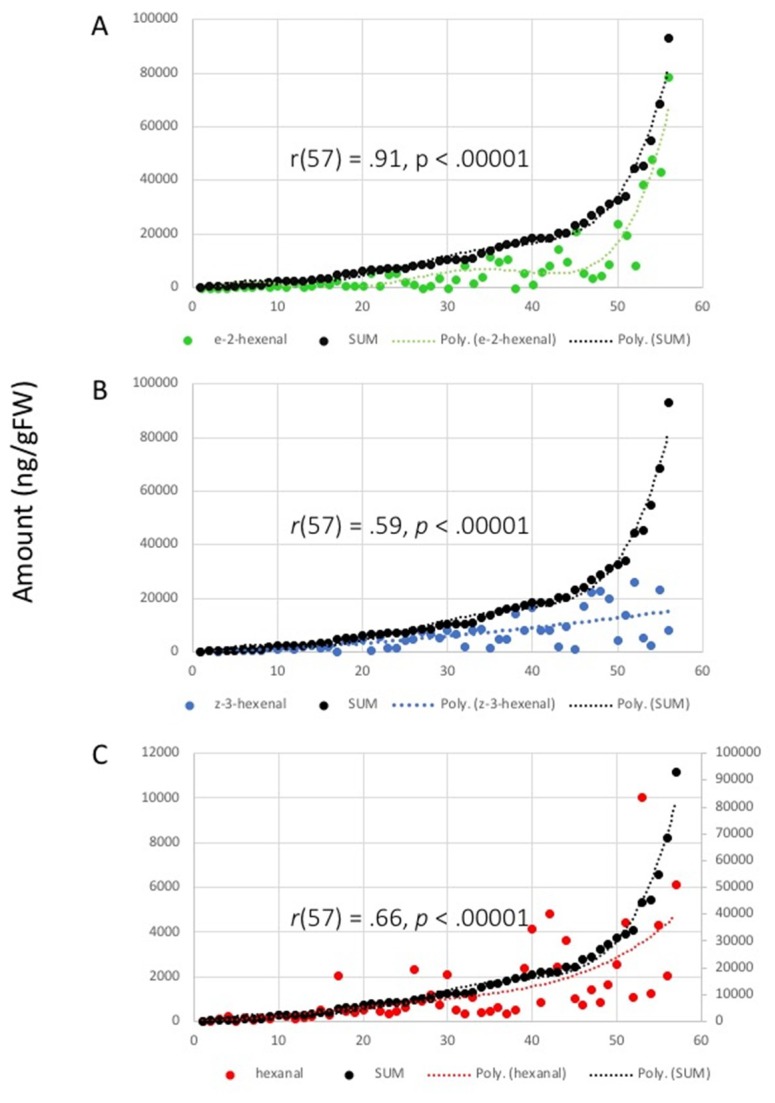
Correlation between total amount of green leaf volatiles and individual compounds after damage. (**A**) correlation of (*E*)-2-hexenal with total amount; (**B**) correlation of (*Z*)-3-hexenal with total amount; (**C**) correlation between hexanal and total amount. Note that, for hexanal (**C**), a separate scale was used due to the large difference in produced amounts. Correlation coefficients (*r*) and *p*-values are shown on the graphs. Trendlines are shown in corresponding colors (dotted lines). Results for each plant are averages from at least three biological replicates.

Surprisingly, a high correlation between E-2-HAL and total amounts was found (Pearson’s correlation coefficient *r* (57) = 0.91, *p* < 0.00001) (Figure 4A). A moderate correlation between Z-3-HAL and total amount was detected (*r* (57) = 0.59, *p* < 0.00001) (Figure 4B). Similar results were found for the correlation between hexanal and total emissions (*r* (57) = 0.66, *p* < 0.00001) (Figure 4C). This suggests that E-2-HAL production is an essential factor for the capacity of plants to release large quantities of GLV.

**Figure 5:**
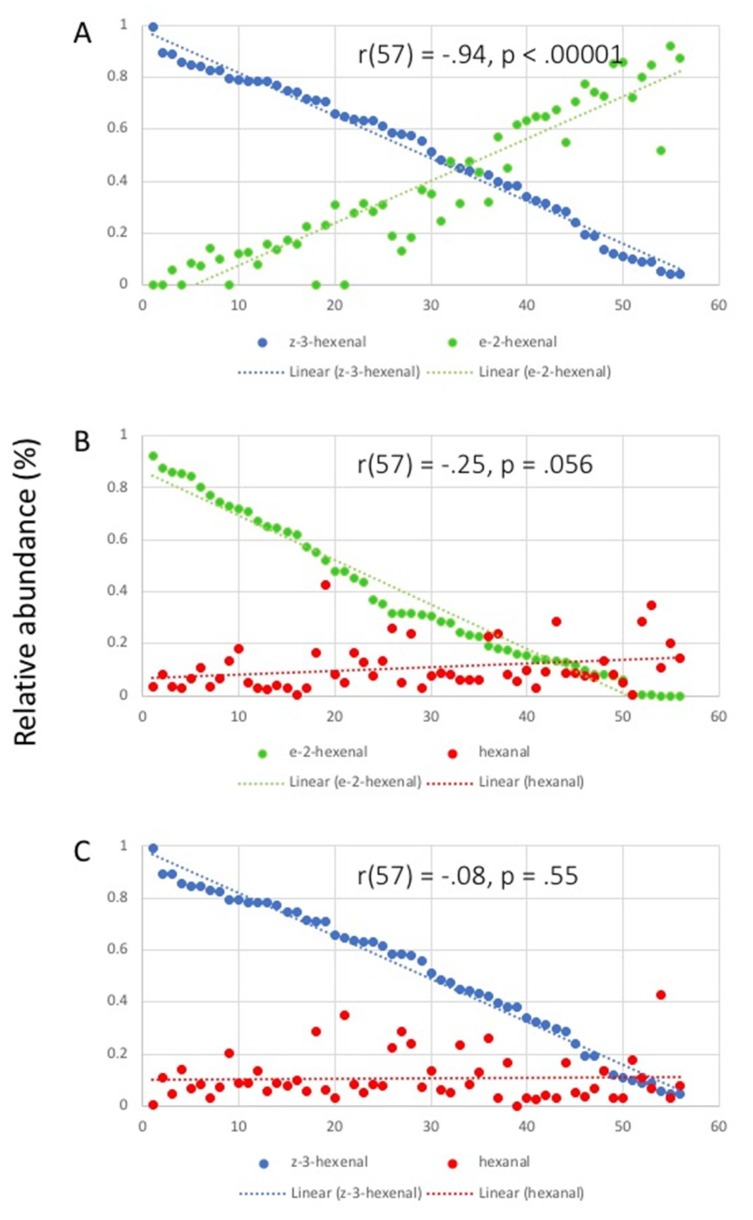
Correlation between relative proportions of individual compounds after damage. (**A**) correlation of (*Z*)-3-hexenal with (*E*)-2-hexenal; (**B**) correlation of (*E*)-2-hexenal with hexanal; (**C**) correlation between (*Z*)-3-hexenal and hexanal. Correlation coefficients (*r*) and *p*-values are shown on the graphs. Trendlines are shown in corresponding colors (dotted lines). Results for each plant are averages from at least three biological replicates.

Secondly, we analyzed the correlation between relative amounts of damage-induced GLV (Figure 5). A strong negative correlation was found for the emissions of E-2-HAL and Z-3-HAL (*r* (57) = −0.94, *p* < 0.00001) (Figure 5A). In contrast, only very weak correlations were found between the emissions for E-2-HAL and hexanal (*r* (57) = −0.25, *p* = 0.056) (Figure 5B), and for Z-3-HAL and hexanal (*r* (57) = −0.08, *p* = 0.55) (Figure 5C). This confirms that the production of E-2-HAL, as expected, comes at the expense of Z-3-HAL.

### 2.3. Damage-Induced GLV Production and Plant Systematics

Our analysis clearly provides evidence for a correlation between E-2-HAL production, overall quantities, and reduction of Z-3-HAL. To further explore correlations between different families, we organized the list of plants, their total GLV release, and the composition of damaged-induced GLV corresponding to the clade system provided by Reference [45]. We did not find any significant correlation between plant families or even a higher order and the release quantities and qualities of GLV (Figure S1, Supplementary Materials) although some trends seem to evolve. For example, within the family of the Curcubitaceae, a high degree of isomerase activity could be detected, while, in the family of Solanaceae, quantities and qualities of E-2-HAL did vary significantly (Figure S1, Supplementary Materials). In contrast, in the Asteraceae family, E-2-HAL was undetectable. However, we did only analyze a maximum of three members for some families, while, for others, only one species was tested. Therefore, the trends seen so far may not be representative for the whole family.

**Figure 6:**
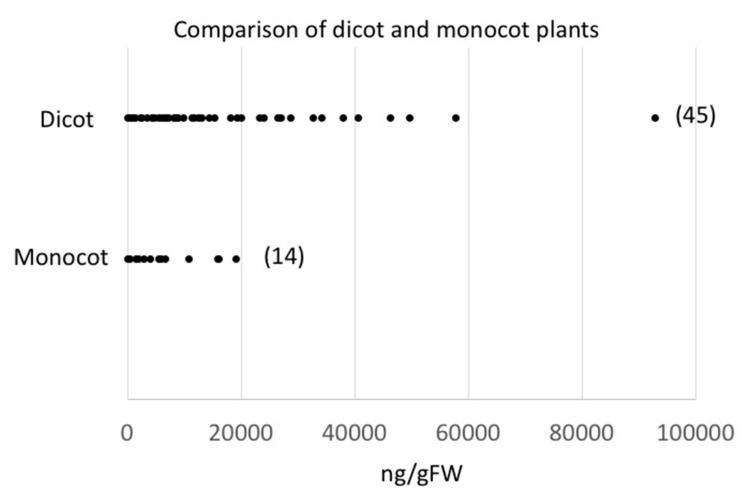
Comparison of green leaf volatile (GLV) release (total amount in ng/g FW) from dicot and monocot plant species. Numbers in brackets indicate the number of plant species tested in each group. Results for each plant are averages from at least three biological replicates.

However, by comparing monocot plants with dicot plants, we found that monocot plants did not produce similar high quantities when compared to peak emitters among the dicot group (Figure 6). However, we only analyzed 14 monocot species (compared to 45 dicots). As described above, these numbers may not be sufficient for a conclusive comparison between the two groups.

## 3. Discussion

The study presented herein is, to the best of our knowledge, the first of its kind. By analyzing 59 plant species representing 32 families in 23 orders, we found a high degree of variation with regard to damage-induced GLV release, both quantitatively and qualitatively. We focused this analysis on the capacity to produce GLV rather than analyzing the responses to herbivory or pathogen infection, as well as abiotic stresses, as described previously [1,2,3,4,5,6,7,8,9,10]. This is mainly because those stresses may individually alter the production of GLV. For example, many insect herbivores modulate GLV emissions through factors abundant in their saliva [41,42,43,44]. Furthermore, cold stress will, as long as its ongoing, likely reduce enzyme activities, including those involved in GLV production [46]. Additionally, intact cells in plants are necessary to convert the aldehydes among the GLVs into the corresponding alcohols and esters [24]. Therefore, the disadvantage of analyzing the qualitative differences of GLV release in the presence of intact cells is that the overall quantities of GLV can vary due to inconsistent damage between samples. In contrast, analyzing the capacity to produce GLV provides us with a baseline that can serve as a starting point when investigating biotic and abiotic factors that can cause the release of these compounds. It may further provide information about what concentrations of GLVs should be used for the treatment of plants by considering what those plants can produce. Likewise, the composition of GLV from different plants must be taken into consideration. These compositions can vary significantly among different plant species, as shown in Figure 1 and Figure 2. To date, very little information is available on how different GLVs may regulate different responses in plants. For maize, we found that all tested GLVs had similar activities with regard to priming herbivore-related defense responses. Likewise, (*Z*)-3-hexenal and (*Z*)-3-hexenyl acetate had similar activities when used to protect plants against cold stress damage. However, for other plants, it was shown that, for example, (*E*)-2-hexenal specifically induced heat stress-related genes, while Z-3-HAL did not [7]. This differential activation of stress-specific genes does, therefore, need to be further investigated, which should be accompanied by an analysis of what that specific plant can produce quantitatively and qualitatively.

More revealing than absolute numbers, which varied significantly, were the correlation analyses. Here, we looked at correlations regarding absolute amounts, as well as proportions, of individual GLVs. We found a high degree of correlation between the total amounts of GLV release and the amount of E-2-HAL in particular. The results suggest that, if individual plant species produce large quantities, an isomerase converting Z-3-HAL into E-2-HAL is essential. This is further supported by our finding that increasing proportions of E-2-HAL are negatively correlated with those of Z-3-HAL, suggesting that the isomerase shifts the equilibrium withing the biosynthetic pathway from Z-3-HAL toward E-2-HAL and, therefore, allows for an overall increase in GLV. It further implies a negative feedback mechanism in the HPL-catalyzed reaction, which may limit the production of GLV. However, the biochemical implications of this finding are yet to be studied. Other factors may further play a significant role in the regulation of GLV production after damage, particularly enzyme abundance, enzyme characteristics, and substrate availability. However, little is known about these aspects of GLV biosynthesis. Most HPLs, for example, seem to prefer acidic pH values for their highest activity [35], which coincides with the pH of damaged plant tissue, while the HPL for spinach (*Spinacea oleracea*) was reported to be more active in basic environments [38]. However, the consequences of these variations in the regulation of HPL for the emission of aldehyde GLV among different plant species are not known.

Likewise, little is known about the abundance of substrates for HPL. While it was assumed that HPLs use free hydroperoxy fatty acids, more recent results showed that certain HPLs use lipid-bound hydroperoxy fatty acids [47]. Furthermore, does the substrate need to be biosynthesized first, which requires an active lipoxygenase withing the damaged tissue, or are substrates readily available upon damage? There is currently very little information available regarding these processes. For maize, it was shown that the lipoxygenase responsible for GLV production (ZmLOX10), while located in the chloroplast, is more active at basic pH [40], while the actual aldehyde GLV production by HPL prefers acidic pH levels. This suggests that substrates for HPL are readily available upon damage, since the lipoxygenase would not be able to produce hydroperoxy fatty acids in this very acidic environment. As for the results shown above, we have basically no information about these factors and the role they play in the release of damage-induced GLV, since only very few plants were functionally studied for their regulation of GLV production.

The organization of the data by phylogenetic principles showed a high degree of variation and did not provide many clues as to what factors may regulate the qualitative and quantitative differences within the different orders used for this study. While this may be due in part to the relatively small sample size, we were nonetheless expecting to find some principles that may help to explain the observed variability. However, we did see a quantitative difference when comparing monocot with dicot plants as shown in Figure 6. Monocot plants that were analyzed for this study were not among the high emitters. This was somewhat surprising since we as humans associate the smell of GLV with freshly cut grass. However, we tested some typical grasses used as turf, but we found them to be rather low emitters compared, for example, to the members of the Curcubitaceae family. Other differences that were observed, like the lack of hexanal emission from members of the Asteracae order, need to be further analyzed, ideally together with an analysis of the fatty-acid composition to confirm our observation, which is currently under investigation in our lab.

To conclude, we showed that the damage-induced release of GLV varies significantly between the different plant species that were selected for this study. Furthermore, while some correlations, both quantitatively and qualitatively, were identified, we were not able to determine principles that provide an underlying system for the release of these common compounds. We, therefore, hypothesize that ecophysiological considerations related to biotic and abiotic stresses may be more relevant for the capacity to produce GLV rather than phylogenetic relationships.

## 4. Materials and Methods

### 4.1. Chemicals

(*Z*)-3-Hexen-1-al (*Z*-3-HAL), (*E*)-2-hexen-1-al (*E*-3-HAL), and dihydrojasmone were purchased from Bedoukian (Bedoukian Research, Danbury, CT, USA). Hexanal was purchased from Sigma Aldrich (St. Louis, MO, USA). All solvents used were analytical grade.

### 4.2. Plant Material

Plants were either grown in the lab under standard conditions (see Table S1) or collected from natural habitats in the vicinity of San Antonio, Texas (TX, United states of America (USA)) over a period of three months (March to May) in 2019. Outside conditions were generally mild with temperatures around 20–25 °C during the day and between 15–20 °C during the night over the collection period. No temperatures below 10 °C were recorded during this time. Overall, we analyzed the release of damage-induced aldehyde GLV from 59 plant species belonging to 32 families in 23 orders. We used fully expanded regular leaves at similar stages. Leaves from at least three individual plants were collected and placed in a plastic bag with a wet paper towel and immediately transported to the lab for analysis. We selected mainly young but fully grown leaves for our analysis. Lab plants (see Table S1, Supplementary Materials) were grown in Ferti-lome Ultimate Potting Mix in a growth chamber under a 12-h photoperiod at 26 °C with 60% relative humidity. Light intensity was set to ca. 150 μmol∙m^2^∙s^−1^. Plants were used at different ages and stages. Leaves from at least three different plants were collected for analysis and immediately analyzed after harvesting.

### 4.3. Leave Damage and Analysis of Green Leaf Volatile Synthesis

To analyze damage-induced GLV emissions, we homogenized 50–200 mg of leaf tissue from individual plants by following the general method of Reference [41] with a few modifications. Tissue (excluding the midvein) was cut from fresh leaves and immediately placed in 2-mL screw-cap centrifuge tubes. Samples were homogenized with Zirmil homogenizing beads (1 mm diameter) in a Precellys tissue homogenizer (MO BIO Laboratories, Carlsbad, CA, USA) at 6000 shakes per minute for 25 s. The 2-mL microcentrifuge tubes were unscrewed without removing the cap and immediately dropped into a 30-mL glass container while also releasing the cap into the glass container to avoid significant losses of volatiles. The glass containers were also immediately capped. Volatile emissions were collected from the tissue homogenate by inserting a volatile collection filter packed with 30 mg of Hayesep Q absorbent (Supelco, Bellefonte, PA, USA) coupled to a vacuum at 0.3 L/min for 1 h, as described previously [6]. Filters were then removed and eluted with 150 µL of dichloromethane, and 1000 ng of internal standard (dihydrojasmone) was added. The analysis of damage-induced GLV release was performed on a Varian 3900 GC coupled to Varian Saturn 2200 MS equipped with split–splitless capillary injector systems in electron impact mode (EI). Injection volume was 1 µL. The data collection, storage, and subsequent analysis were performed on a computer using the Varian MS Workstation software. Helium at a constant flow rate of 1 mL/min was used as a carrier gas. The analyses of volatiles were performed on a fused silica capillary column (Equity™ 30 m × 0.25 mm inner diameter with a 0.25-µm-thick film of bonded methyl silicone). The GC was programmed as follows: 40 °C for 2 min, then 15 °C/min to 250 °C. All of the injections were made in the split mode (1:20 split ratio). Compounds were identified by comparison to authentic standards (retention time and fragmentation).

### 4.4. Phylogenetic Analysis

The linear listing of studied plant species (Figure S1, Supplementary Materials) followed the sequence of plants by taxonomic order and advocated by the Angiosperm Phylogeny Group (APG) III system [45]. For an easier overview, the traditional distinction between monocots and dicots was used for Figure 6.

### 4.5. Statistical Analysis

At least three biological replicates were performed per plant. Averages and standard deviations were calculated for each plant (Table S1, Supplementary Materials). We further used correlation analysis for comparisons. Pearson’s correlation coefficients and *p*-values were calculated in Microsoft Excel to analyze statistical relationships between individual components and specific amounts of the respective GLVs.

## Supplementary Materials

The following are available online at https://www.mdpi.com/2223-7747/9/2/213/s1, Table S1: Green leaf volatile release by different plant species; Figure S1: Systematic analysis of green leaf volatile release.
